# Supplementary material for: A functional topography within the cholinergic basal forebrain for encoding sensory cues and behavioral reinforcement outcomes
Source: eLife. 2021 Nov 25;10:e69514. doi: 10.7554/eLife.69514 (PMC8654357; doi:10.7554/eLife.69514)
Supplement: Transparent reporting form [file elife-69514-transrepform1.docx]

***eLife’s* transparent reporting form**

We encourage authors to provide detailed information *within their submission* to facilitate the interpretation and replication of experiments. Authors can upload supporting documentation to indicate the use of appropriate reporting guidelines for health-related research (see [EQUATOR Network](http://www.equator-network.org/%20)), life science research (see the [BioSharing Information Resource](https://biosharing.org/)), or the [ARRIVE guidelines](http://www.plosbiology.org/article/info:doi/10.1371/journal.pbio.1000412) for reporting work involving animal research. Where applicable, authors should refer to any relevant reporting standards documents in this form.

If you have any questions, please consult our Journal Policies and/or contact us: [editorial@elifesciences.org](mailto:editorial@elifesciences.org).

**Sample-size estimation**

- You should state whether an appropriate sample size was computed when the study was being designed
- You should state the statistical method of sample size computation and any required assumptions
- If no explicit power analysis was used, you should describe how you decided what sample (replicate) size (number) to use

Please outline where this information can be found within the submission (e.g., sections or figure legends), or explain why this information doesn’t apply to your submission:

We did not perform a power analysis to identify a target sample size for this study. We identified a target sample size based on previous publications using similar methods (Cai et al., eLife 2020, Crouse et al., eLife 2020).

**Replicates**

- You should report how often each experiment was performed
- You should include a definition of biological versus technical replication
- The data obtained should be provided and sufficient information should be provided to indicate the number of independent biological and/or technical replicates
- If you encountered any outliers, you should describe how these were handled
- Criteria for exclusion/inclusion of data should be clearly stated
- High-throughput sequence data should be uploaded before submission, with a private link for reviewers provided (these are available from both GEO and ArrayExpress)

Please outline where this information can be found within the submission (e.g., sections or figure legends), or explain why this information doesn’t apply to your submission:

For each analysis of fiber-based calcium imaging, we performed 11 biological replicates (one sample per mouse, 11 mice). For each analysis of fiber-based ACh sensor imaging, we performed 10 biological replicates (one sample per mouse, 10 mice).

The numbers of technical replicates are detailed in the Methods section. Analysis was performed on individual trial data. During the sensory characterization session, each unique trial type was repeated 20 times each (Figure 2). During behavioral training (Figs 1, 3-6), any given measurement condition was dictated by the animals’ behavior. Technical replicates were collected over 10 behavioral sessions and generally produced over 100 replications for any unique measurement. The timeline of the experiment is shown in Figure 2A.

We did not exclude any subjects from the analysis of fiber photometry data. We did not exclude any trials from the analysis of fiber photometry data. For the analysis of the size of the pupil, we excluded four mice for which the algorithm could not accurately identify the pupil over the full session (Figures 1F, 1G and 2E). This is reported on lines 811-813.

**Statistical reporting**

- Statistical analysis methods should be described and justified
- Raw data should be presented in figures whenever informative to do so (typically when N per group is less than 10)
- For each experiment, you should identify the statistical tests used, exact values of N, definitions of center, methods of multiple test correction, and dispersion and precision measures (e.g., mean, median, SD, SEM, confidence intervals; and, for the major substantive results, a measure of effect size (e.g., Pearson's r, Cohen's d)
- Report exact p-values wherever possible alongside the summary statistics and 95% confidence intervals. These should be reported for all key questions and not only when the p-value is less than 0.05.

Please outline where this information can be found within the submission (e.g., sections or figure legends), or explain why this information doesn’t apply to your submission:

Details of the statistical analyses are given in the main manuscript and figure legends. Each reference to a statistical test is accompanied by the sample size and appropriate metrics.

(For large datasets, or papers with a very large number of statistical tests, you may upload a single table file with tests, Ns, etc., with reference to sections in the manuscript.)

**Group allocation**

- Indicate how samples were allocated into experimental groups (in the case of clinical studies, please specify allocation to treatment method); if randomization was used, please also state if restricted randomization was applied
- Indicate if masking was used during group allocation, data collection and/or data analysis

Please outline where this information can be found within the submission (e.g., sections or figure legends), or explain why this information doesn’t apply to your submission:

This study included a single experimental group. The assignment of tone frequency to reinforcer type was randomized and balanced across mice.

**Additional data files (“source data”)**

- We encourage you to upload relevant additional data files, such as numerical data that are represented as a graph in a figure, or as a summary table
- Where provided, these should be in the most useful format, and they can be uploaded as “Source data” files linked to a main figure or table
- Include model definition files including the full list of parameters used
- Include code used for data analysis (e.g., R, MatLab)
- Avoid stating that data files are “available upon request”

Please indicate the figures or tables for which source data files have been provided:

The source data files in a *.mat* format and the Matlab code to reproduce the produce Figures 1-7 and their supplements are available on Mendeley Data (Robert B, Kimchi, EY, Watanabe Y, Chakoma T, Jing M, Li Y, Polley DB | 2021 | “Data for: "A functional topography within the cholinergic basal forebrain for encoding sensory cues and behavioral reinforcement outcomes."” | dx.doi.org/10.17632/d8tjdxyjcm.1|Mendeley Data, doi: 10.17632/d8tjdxyjcm.2).

We included a spreadsheet with the details of the quantification of immunolabeled cells shown in Figure 1D in Figure 1 – Source Data 1.
